# The role of pyrethroid derivatives in autophagy and apoptosis crosstalk signaling and potential risk for malignancies

**DOI:** 10.18632/oncotarget.28328

**Published:** 2022-12-17

**Authors:** Jyothi Puvula, Narendra Maddu, Nagajothi Gutam, Asha Parimal, Pongali B. Raghavendra

**Affiliations:** ^1^Department of Biochemistry, Sri Krishnadevaraya University, Anantapuramu 515003, Andhra Pradesh, India; ^2^Department Corporate Secretaryship-Biostatistics, Queen Mary’s College, Chennai 600004, Tamil Nadu, India; ^3^School of Regenerative Medicine (SORM) - Manipal Academy of Higher Education, Deemed to be Manipal University, Bangalore 560065, Karnataka, India; ^4^National Institute of Biomedical Genomics, Kalyani 741251, West Bengal, India; ^*^These authors contributed equally to this work

**Keywords:** allethrin, prallethrin, autophagy, apoptosis, Ccl2

## Abstract

Pyrethroids and its derivatives widespread and uncontrolled continuous use has influenced multiple deleterious effects resulting in as a potential risk factor causing damage to the organ systems. Allethrin and prallethrin are extensively used yet their influences on human primary cells are very limited or under reported. The potential mechanisms by which allethrin and prallethrin modulates human primary cells, especially the molecular mechanisms or interconnectivity of autophagy-apoptosis, their clinical relevance in human subjects or patients are not well defined. In this current study, we've furnished the evidence that both allethrin and prallethrin user samples significantly induced Ccl2 mRNA expression, increased amount of reactive oxygen intermediate, inhibited membrane bound enzymes and altered membrane fluidity. Pyrethroid derivative users had induced levels of lipid peroxidation and induced binding activities of transcription factors(tfs) like CEBP-β and NF-AT. Pyrethroid derivatives induced autophagy, elicited intracellular Ca2+ concentration, calcineurin and regulated proapoptotic genes, DAPK1, Bim. Our current study presumably comprises the initial investigation of a very new mechanism of pyrethroid derivatives-moderated programed cell death in various cell sets or types, like human primary cells where-in this is a late event, is documented. Hence, current research-study might be significant in the various pyrethroid derivatives-allied hematological-related cancers and immunosuppressant or auto-immune disorders. In the foremost instance, we present data stating that pyrethroid derivatives induces multiple cell signaling cascades, like CEBP-β, NF-AT, ERK and MAPK having a role in autophagy thereby; synchronously effectively impact on the apoptosis, therefore causing hematological tumors and toxic or immune related disorders.

## INTRODUCTION

Pyrethroids are extensively used insecticides by virtue of insecticidal activity potential in Asia especially, India and in different nations or worldwide to get safety-defense to counter mosquitoes and insects for diverse household or the agricultural needs [1–11]. Pyrethroids are applied for household pest control and they are mostly noticed in a various commodities, which includes like household or garden insecticides, sprays, shampoos, lice treatments, and repellents. Over the half of world population have adopted pyrethroid insecticides utility that might attribute surpassingly 25% of insecticide trade of industrial nations in 90’s and their usage quantum have been increasing now in most of these countries, [12, 13] as ubiquity of mosquitoes and related insects are more in most of the endemic places of the globe. Primarily, pyrethroids were considered as vastly lethal to the insects and lower toxic to the humans [14]. Pyrethroid instigate neuro related toxicity and deleterious health impacts, exposure might weaken the neurodevelopment, impede with the reproductive health, and augment threat of significant chronic diseases, such as COPD, diabetes, cardiovascular disease (CVD) and Parkinson disease spanning from entire body upheaval to clamors and later to death are further established [13, 15–20]. Very limited published data is available up to this point on the impacts of pyrethroids on humans, and steadily the associated facts of their toxicity are impending into light. So the repercussions of persistent exposure to pyrethroids on lengthy haul health events in the humans remains to be established. The mortality rate of pyrethroids exposure in India in recent years have been reported to be in rise from 12.5 to 25% [21]. Allethrin and prallethrin are amidst the utmost extensively adopted pyrethroid insecticides. Allethrin poisoning might be noticeably larger persistent because of its tranquil availability as repellent for mosquitos and the sprays-insecticidal etc., [22] frequent reporting of pyrethroid poisoning in India are evident [23, 24]. Comprehensibly nil pertinent data on awful-chronic effects related to pyrethroid toxicity exist in open scientific literature for humans and animals [21, 25]. Eventually the utility of all these pyrethroids is habitually or commonly as repellents mainly for the mosquitos, also as sprays for agriculture or horticulture needs wherein people continuously exposure or the compounds inhalation for prolonged durations, impending awful utility stir up a worry amid public currently, which initiated the ground layout of the current research study. Pyrethroids derivatives persistent exposures are termed to be neurotoxic. Numerous tumor related immune or lung disorders contributing to lowering of immunity in humans have been reported. Exposures to these derivatives also lead in the reduction of IgG immunoglobulin’s concentration. Pyrethroids derivative’s also instigate the allergies and asthma exceptionally the occupational pesticide exposure may influence respiratory health, since it is associated with surge odds of wheezing, coughing and asthma related respiratory or lung ailments, its immunosuppressive effects can diminish host resistance against infections. Exposure to all such compounds can also contribute to induction of the hematopoietic or inflammatory disorders, especially in patients with impaired immune function. The objective of the current-study is twofold; Primarily, to determine the alterations in membrane fluidity of human platelets (PRP) derived from volunteers exposed to habitual usage of allethrin and prallethrin, to comprehend the function and level of Na^+^/K^+^-ATPase activity and Ca^2+^-dependent ATPase activity and Mg^2+^-ATPase in allethrin and prallethrin analogous users and secondarily is to understand the impact of pyrethroid derivatives mediated autophagy and apoptosis in human primary cells. Autophagy is beneficial to the cells and individuals, which includes the consecutive series of events inclusive of formation of double membrane, elongation, vesicle maturation and eventually conveyance of the targeted components to the lysosome. It also prompts to maintain cellular homeostasis and is crucial in a broad spectrum of conventional human physiological processes [26]. However, an amplifying number of disorders are connected to the dysregulated autophagic process. Pyrethroids derivatives mediated deleterious effects which includes acute chronic inflammation, apoptosis, different blood mediated cancers, respiratory ailments and autoimmune related disorders. Overall this current study might facilitate to formulate therapeutics or intervention targets that might serve to decrease the effect or impact of pyrethroids derivatives by targeting the signaling cascade that serves to minimize the modulation of autophagy mediated apoptosis.

## RESULTS

### Modulation of cytokine, chemokines and ROS levels by pyrethroids

**Figure 1:**
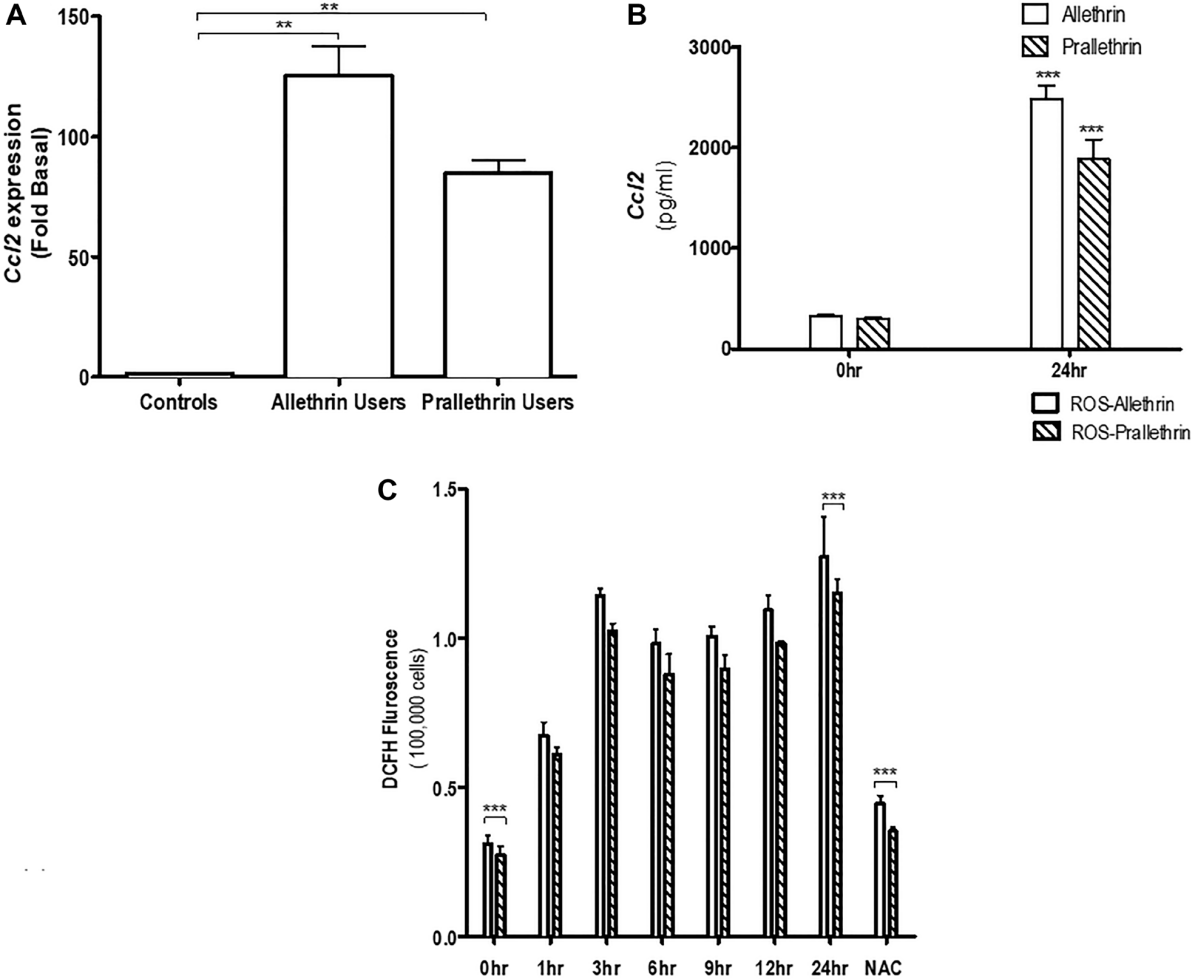
The effect of pyrethroid derivatives on CCL2 expression and ROS response. (**A**) Effect of Allethrin and Prallethrin on Ccl2 expression and production in the allethrin and prallethrin derivative exposure users or treatments and healthy controls derived from human PBMCs (peripheral blood derived monocytes) and THP-1 cells: Cells were washed with PBS and allowed to rest 24 h. RNA was extracted and mRNA levels determined using quantitative real-time RT-PCR as described before (Raghavendra et al. 2013). (**B**) PBMCs were treated with Allethrin (100 μM), or Prallethrin (100 μM) for 24 h. Supernatants were analyzed for CCL2 levels using ELISA from Biosciences Inc, as described before (Raghavendra et al. 2013). (**C**) Human THP-1 cells were treated with Allethrin (100 μM), or Prallethrin (100 μM), DMSO (100 μM) and N-acetylcysteine, a well-known free radical scavenger - NAC (10μM) for 0, 1, 3, 6, 9, 12 and 24 h. ROS associated fluorescence levels were measured kinetically as described in the methods. ^*^ *p* < 0.05; ^**^ *p* < 0.01. *N* = 3–10.

We first investigated the influence of Allethrin and Prallethrin on different chemokines expression or modulation levels of in Allethrin and Prallethrin users and healthy controls derived human PBMCs. Interestingly, both Allethrin and Prallethrin user samples significantly induced Ccl2/MCP-1 (Monocyte chemoattractant protein-1) mRNA expression over healthy controls, (Figure 1A) but had no effect in both Ccl1 or Ccl5 (data not presented). This effect was scrupulous for Ccl2 in the Allethrin and Prallethrin users and had no affect with different chemokines (data not presented). We later used the culture supernatants and measured for CCL2 levels. Both Allethrin and Prallethrin treatment were capable to singly enhance CCL2 production as quantified in the culture supernatants (Figure 1B). Collectively, our findings showed differential effects of Allethrin and Prallethrin users or treatments on the cytokine and chemokine expression levels. We also have shown that with Allethrin and Prallethrin treatment and incubation in human PBMCs cells in a time-dependent fashion resulted to form the reactive oxygen species (ROS). The Mito-SOX and dihydroethidine dyes were used to reveal the presence of Allethrin and Prallethrin-mediated superoxide anions. NAC also showed to block ROS formation (Figure 1C). Together summing, this data implies that exposure of pyrethroid derivatives in the human cells resulted in the generation of superoxide anion radicals that moderate cell damage.

### Pyrethroids inhibited membrane bound enzymes and altered membrane fluidity in human platelet membrane

**Figure 2:**
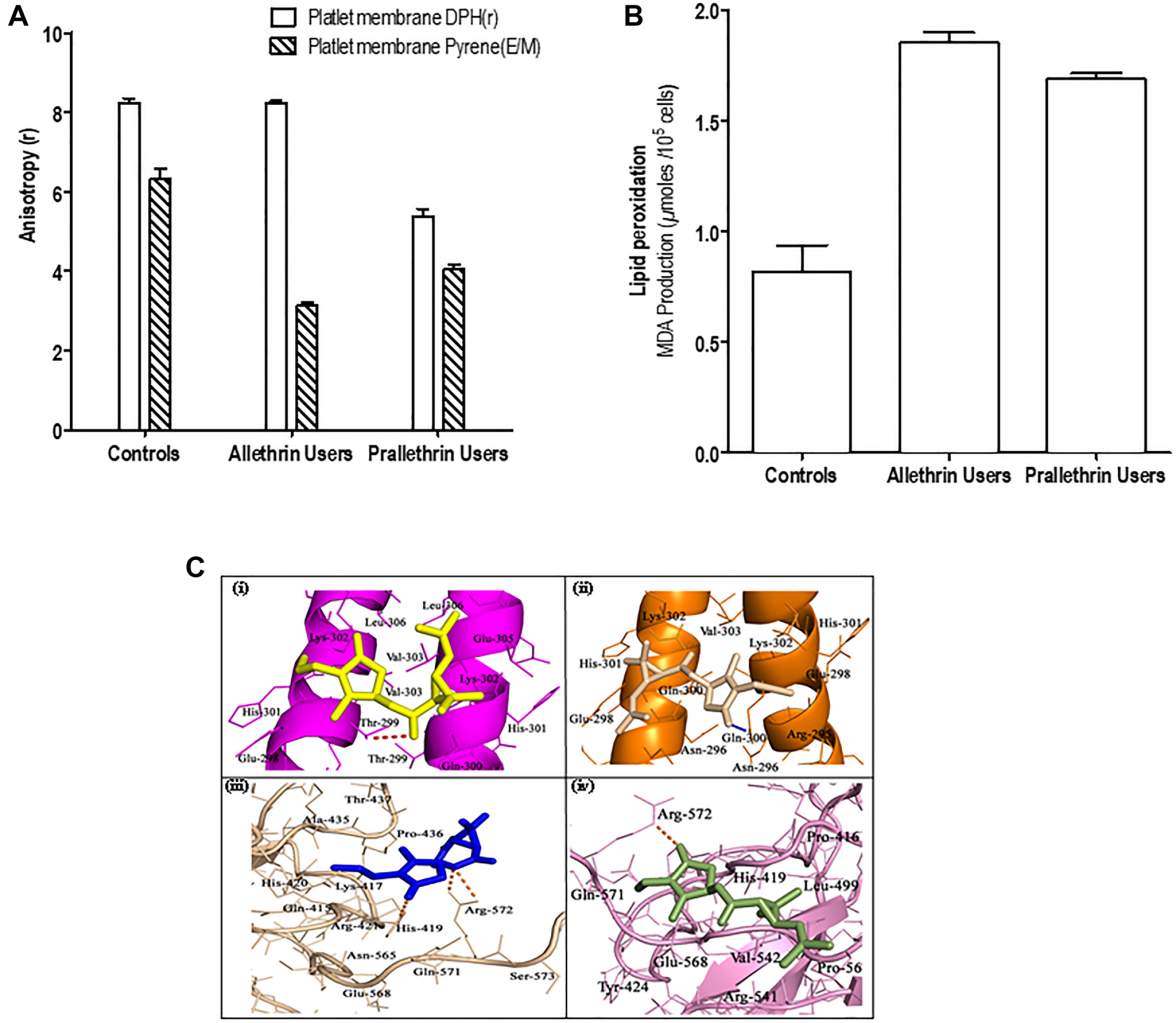
The effect of pyrethroid derivatives on platelet membrane, lipid peroxidation and DNA binding. (**A**) Effect of Allethrin and Prallethrin on platelet membrane. Two milliliters of samples in PBS was added to 2 μl of DPH solution (190 mM) in a cuvette and the fluorescent anisotropy was measured after incubation at 37°C for 20 min. The values denote mean ± S.D. independent determinations of *N* = 3. (**B**) Effect of Allethrin and Prallethrin derivative exposure users and healthy control on lipid peroxidation. Similarly, samples were used and lipid peroxidation was assayed by measuring malondialdehyde as represented. The values denote mean ± S.D. independent determinations of *N* = 3. (**C**) Docking interaction of Allethrin and Prallethrin with transcription factors CEBP-β (PDB id: 1GU4) and NFAT (PDB id: 2O93) was performed with the AutoDock4.0 program.

In noting the importance of Na^+/^K^+^-ATPase in tuning of intracellular Ca^2+^level and pyrethroids capability to enhance intracellular free Ca^2+^level, the impact of enzymes -Total ATPase, Na^+^/K^+^-ATPase, Mg^2+^-ATPase, Ca^2+^-ATPase activity was tested. Platelet membrane bound enzymes levels were notably reduced and were noticed for Total ATPase, Na+/K+-ATPase, Mg2+-ATPase, Ca2+-ATPase in the platelets of allethrin and prallethrin exposed subjects group II and group III - as in the (Table 1), with contrast for controls group I, results are shown via the (Table 2). To access the role or mechanisms of pyrethroids-associated impediment of Na+/K+-ATPase and other membrane bound enzymes or protein, quantified the membrane fluidity by measuring diphenylhexatriene (DPH) binding or pyrene amidst membrane lipids (Figure 2A). As noticed the DPH binding, with the fluorescence spectrum, lowered in pyrethroid derivative users to controls suggesting that pyrethroids alter membrane fluidity.

**Table 1:** Clinical characteristics of pyrethroid derivatives: allethrin or prallethrin users and healthy control subjects

Parameter	Groups		
	Control	Allethrin users	Prallethrin users
Total Number	10	10	10
Height (cm)	160.93 ± 5.04	161.98 ± 4.99	161.27 ± 6.55
Weight (kg)	51.91 ± 4.35	53.00 ± 5.11	54.75 ± 6.15
BMI (kg/m^2^)	20.44	20.06	20.32
Platelet Count (Mean ± SD) 10^5^ cells/μl	258 ± 59	230 ± 61	234 ± 64
Below 1 × 10^5^ cells/μl	None	17	18
Above 2 × 10^5^ cells/μl	None	3	2
Percentage platelet aggregation when compared to age and sex matched normal volunteers 80%	None	2	3
Percentage platelet aggregation when compared to age and sex matched normal volunteers 50%	None	18	17

**Table 2:** Activity of total Na^+^/K^+^, Mg2^+^ and Ca2^+^ ATPase’s in the platelet membrane of allethrin or prallethrin users and healthy control subjects

Parameter	Groups		
	Controls	Allethrin users	Prallethrin users
Total ATPase	89.31 ± 2.15a	68.79 ± 3.68b	67.13 ± 3.60b
Na^+^/K^+^-ATPase	27.07 ± 1.04a	21.91 ± 1.16b	20.57 ± 0.57b
Mg2^+^-ATPase	43.67 ± 1.00a	34.22 ± 0.74b	34.11 ± 0.82b
Ca2^+^-ATPase	40.18 ± 1.30a	28.66 ± 0.97b	28.25 ± 1.59b

Activities are expressed as nmoles of phosphorus liberated/min per 1 × 10^5^ platelets. Values are expressed as mean ± SEM in each column, followed by the same letter are not significantly different (*P* ≤ 0.05) from each other according to Duncan’s Multiple Range (DMR) test, *N* = 10.

### Pyrethroids effect on the lipid molecules derived from platelet membrane

The results obtained from this showed a prominent increment in the membrane cholesterol (C) and diminish in phospholipid (P) contents besides nil major difference in the protein constituents in platelet membranes of allethrin and prallethrin exposed subjects group II and group III rather in contrast to the controls group I. Further two fold enhancement was seen in subsequent cholesterol to phospholipid (C: P) proportion or ratio in allethrin and prallethrin subjects exposure contrasted for controls the results are represented in the (Table 3).

**Table 3:** Levels of phospholipid/cholesterol and cholesterol/phospholipid (c: p) ratio in the platelets of allethrin or prallethrin users and healthy control subjects

Parameter	Groups		
	Controls	Allethrin users	Prallethrin users
Cholesterol (ng/1 × 10^5^ platelets)	0.162 ± 0.009a	0.212 ± 0.009b	0.240 ± 0.006b
Phospholipid (ng/1 × 10^5^ platelets)	0.204 ± 0.009a	0.140 ± 0.006b	0.137 ± 0.005b
Platelets Cholesterol/Phospholipid ratio	0.640a	1.222b	1.537b

Values are expressed as Mean ± SEM, in each column, followed by the same letter are not significantly different (*P* ≤ 0.05) from each other according to Duncan’s Multiple Range (DMR) test, *N* = 10.

### Pyrethroids induces lipid peroxidation

Lipid peroxidation - a well-known marker for apoptosis, the impact of pyrethroids was studied on lipid peroxidation in the human platelets (*PRP*) by measuring levels of malondialdehyde (MDA) production (Figure 2B). Pyrethroid derivative users had induced levels of lipid peroxidation over the controls platelets.

### Pyrethroids effect on CEBP-β and NFAT binding activities

All physicochemical properties like TPSA (Polar surface area), molecular weight-(mw), logP (Octanol-water partition coefficient), H-bond donors/acceptors and manifold of rotatable bonds of the compounds calculated *in-silico* are outlined in (Supplementary Tables 1–3). *In-silico* physicochemical properties of ligands were assessed as per Lipinski’s Rule of Five [27] using molinspiration software. ADMET properties for compounds were estimated employing the pre-ADMET server (http://preadmet.bmdrc.org/). Also using this server, ADMET properties of plasma protein binding *in vitro*, MDCK cell permeability *in vitro*, absorption of human intestinal, BBB penetration *in vivo*, Caco-2 cell permeability *in vitro* and Pgp-impediment predicted studies are documented. All the predicted ADME features for ligands have shown significant values. *In-silico* toxicity results indicated both compounds exert mutagenic activity in Ames test and they have also shown carcinogenic activity on experimental rats. Docking performance for target proteins with lead molecules were carried out by autodock4. Fifty (50) docked confirmations collected for individual ligand and validation with the elevated docking energy values were chosen to the same degree for optimal binding confirmation. Best docking scores (Kcal/mol) along with inhibition constants (Ki) obtained for allethrin and prallethrin with target proteins are shown in (Supplementary Table 3). The active site residues involved in binding interactions with ligand molecules are represented in (Figure 2C). Thr-299 of the CEBP-β and Arg-572 and His-419 of NFAT are formed H-bond interactions with allethrin compound, whereas prallethrin can formed H-bond interactions with Gln-300 (CEBP-β receptor) and Arg-572 (NFAT protein). Other neighbouring amino acid residues for ligands of target proteins are established hydrophobic and Van der Waals forces.

### Pyrethroids enhanced autophagy

**Figure 3:**
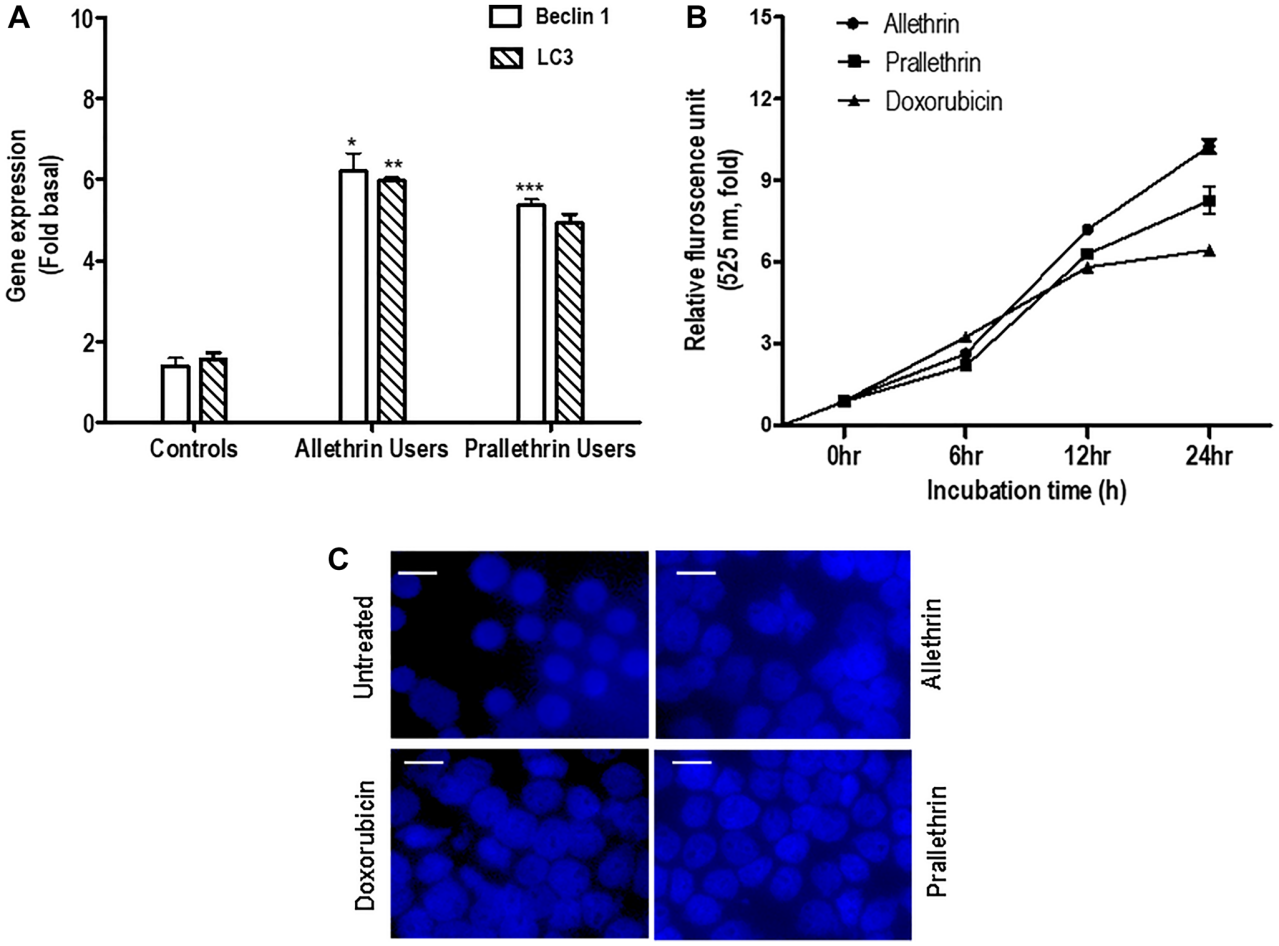
The effect of pyrethroid derivatives on induction of autophagy. (**A**) RT- PCR analysis of autophagy genes in allethrin and prallethrin exposure users and healthy control PBMCs cells. Cells were washed with PBS and allowed to rest 24 h. RNA was extracted and mRNA levels determined using quantitative real-time RT-PCR as described before for Beclin-1 and LC3 genes, (Raghavendra et al. 2013). (**B**) PBMCs were treated with Allethrin (100 μM), or Prallethrin (100 μM), Doxorubicin (100 μM) and DMSO (100 μM) for 0, 6, 12 and 24 h in triplicates. After treatments, cells were fixed with paraformaldehyde (4%), washed three times with PBS, stained with MDC (50 μM) for 15 min, and washed three more times with PBS. Cells were collected, and fluorescence was measured and indicated as–fold, considering the unstimulated cell value as 1-fold from three independent experiments. (**C**) Cells were stimulated with Allethrin, Prallethrin, and Doxorubicin for 24 h and stained with Monodansylcadaverine (MDC) staining. The fluorescence of stained cells was visualized under a fluorescence microscope (scale bar, 10 μM). Error bars represent mean ± S.E. (Student’s *t* test). ^*^ *p* < 0.05; ^**^ *p* < 0.01; ^***^ *p* < 0.001.

The autophagy markers (LC3B, a known indicator of autophagy induction; and Beclin1, proteins as autophagy initiation) were determined in Allethrin and Prallethrin user derived primary cells. PBMCs cells derived from allethrin and prallethrin user samples significantly induced Beclin 1 and LC3 mRNA expression over healthy controls. (Figure 3A). Further to explore the impact of pyrethroids on autophagy, THP-1 cells were treated with Doxorubicin, Allethrin or Prallethrin for different time points. Then MDC was used to stain the cells, the fluorescence intensity was quantified from three independent experiments and denoted in-fold of induction. Autophagy in a dose-dependent manner was estimated by fluorescence photometry (Figure 3B). All the three activators enhanced the MDC fluorescence kinetically, besides allethrin and prallethrin-associated upregulation was greater compared with doxorubicin. THP-1 cells stained with MDC continuously exhibited enhanced fluorescence intensity due to the result of autophagosome formation by allethrin and prallethrin as shown in (Figure 3C).

### Pyrethroids induces intracellular Ca^2+^ concentration and calcineurin activity

**Figure 4:**
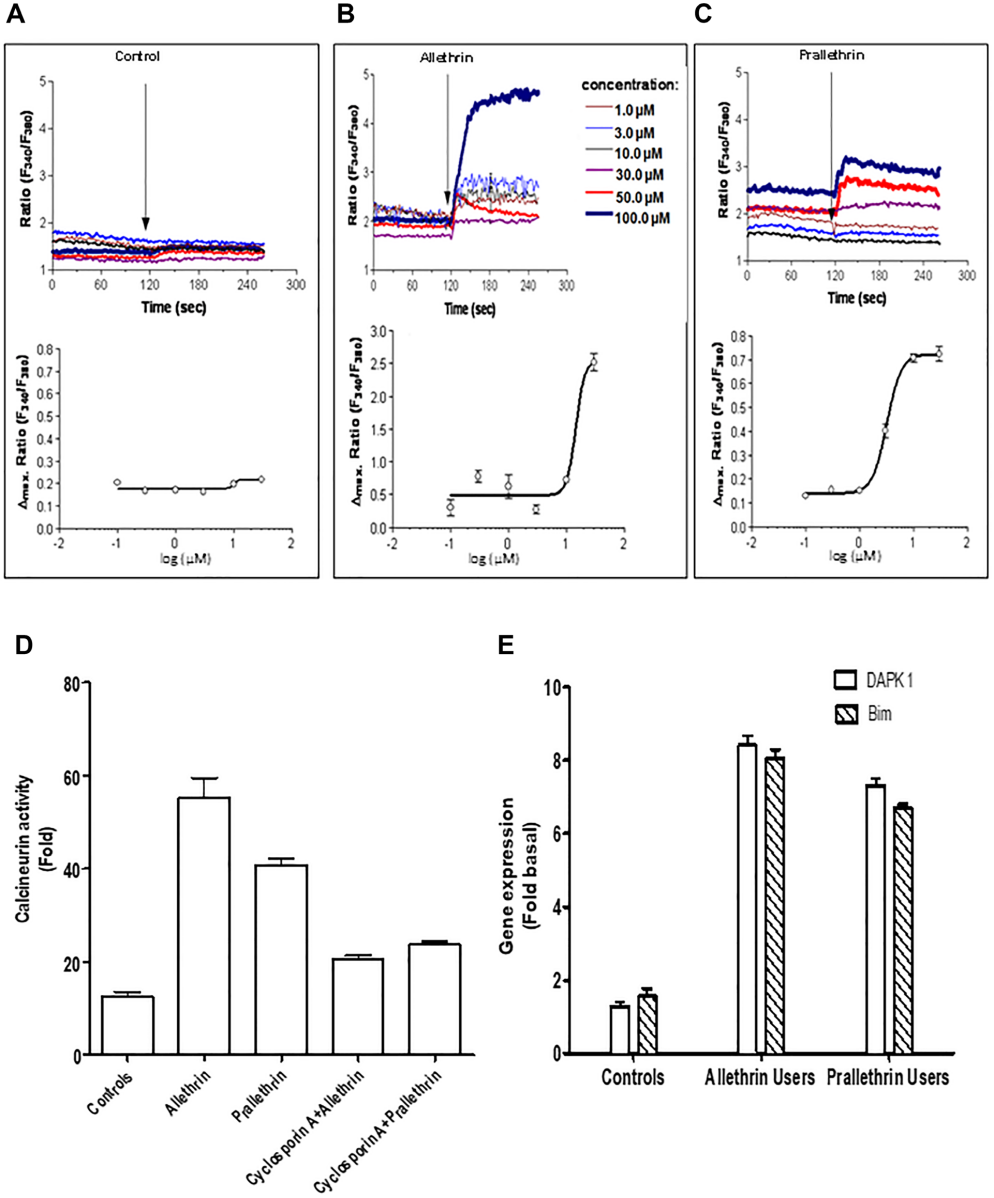
Pyrethroid derivatives elicited intracellular Ca2+ concentration, calcineurin and regulated proapoptotic genes, DAPK1/Bim. (**A**–**C**) Fura-2/AM-loaded cells were resuspended either in Ca2+-containing buffer or Ca2+-free buffer containing EGTA. The ratio of fura-2 fluorescence intensity at the two excitation wavelengths (340/380 ratio) was monitored spectrophotometrically in a stirring cuvette during exposure to different concentrations ranging from 1μM - 100 μM Allethrin, Prallethrin. Results are representative single traces out of four experiments performed in duplicates. (**D**) THP-1 cells were treated with Allethrin (100 μM), or Prallethrin (100 μM) for 24 hr and calcineurin activity was assayed from cell extracts. Cells extract from Allethrin (100 μM), or Prallethrin (100 μM)-treated (24 h) cells was incubated with Cyclosporin A (2.5 μM) and calcineurin activity was assayed. (**E**) RT- PCR analysis of cell death genes in allethrin and prallethrin exposure users and healthy control PBMCs cells. Cells were washed with PBS and allowed to rest 24 h. RNA was extracted and mRNA levels determined using the quantitative real-time RT-PCR as described before for DAPK1 (Calcium/calmodulin–regulated (CaM-regulated) and Bim (Bcl-2-interacting modulator of cell death) genes.

Pyrethroid derivatives interacted with platelet membranes then altered membrane bound enzymes and membrane fluidity. Hence this prompted us to study the intracellular free Ca^2+^ levels and the calcium-dependent serine-threonine phosphatase, calcineurin activity. Calcineurin activity in PBMC cells was increased to almost 50 fold in pyrethroid derivative users to controls (Figure 4D). Also increased intracellular Ca^2+^ level with pyrethroid derivatives for different treatments in human derived cells over controls was detected by Fura-2AM. (Figure 4A–4C). These data suggest the role pyrethroid derivatives users or treated cells for Ca2^+^or calcineurin activity as indicated.

### Pyrethroids increases the pro-apoptotic genes expression, DAPK1 and BIM

We focused to examine the calcium/calmodulin-regulated (CaM-regulated) protein kinase gene so we studied, DAPK1 gene that activates death signaling and also considered BIM gene, BCL-2 protein family’s pro-apoptotic member that behaves as an apoptotic activator. Human DAPK1 and BIM gene expression levels normalized with HPRT were increased in pyrethroid derivative users to controls cells suggesting that pyrethroids derivatives enhanced levels of pro-apoptotic genes as shown in the (Figure 4E).

### Pyrethroids mediated autophagy and effect of inhibitors

**Figure 5:**
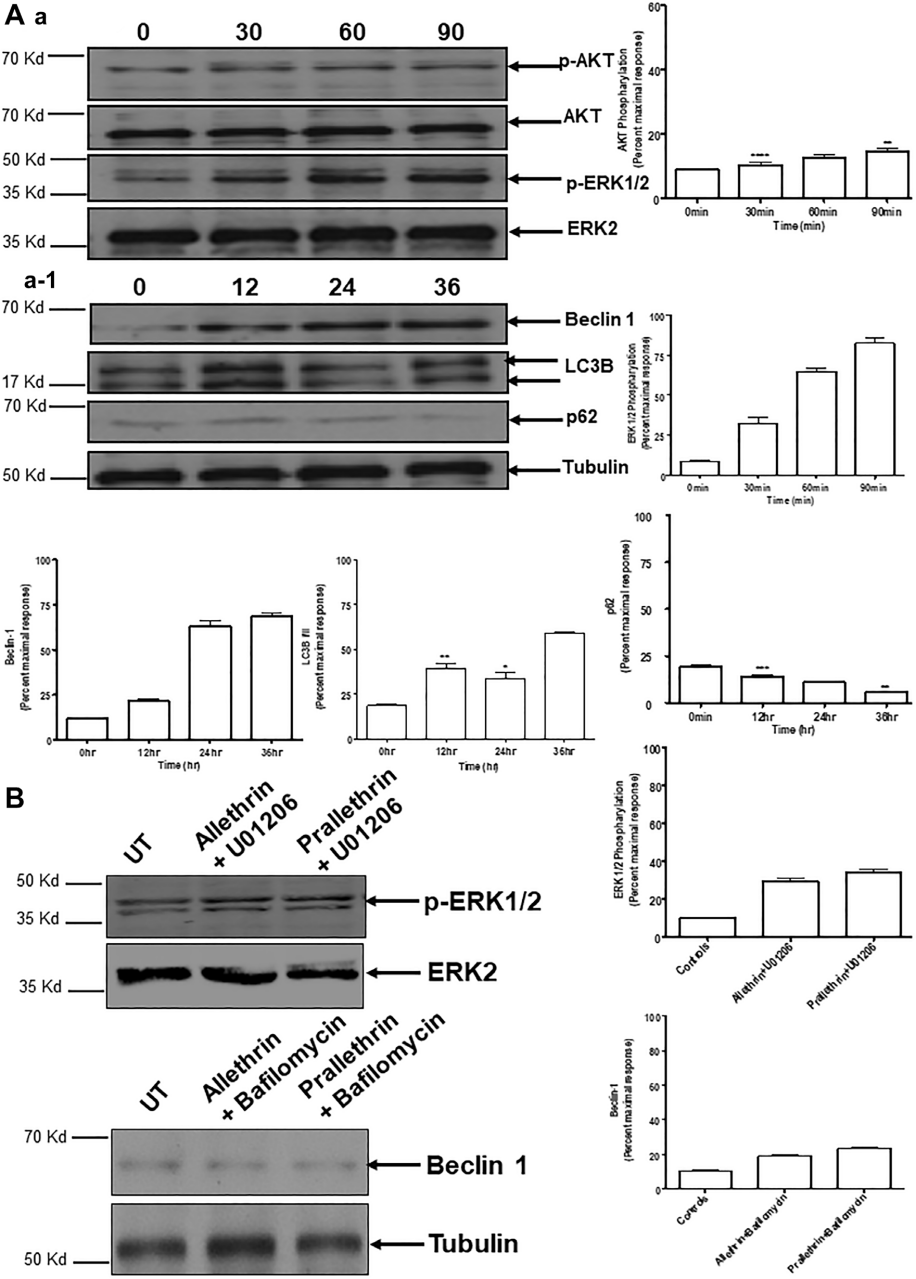
Effect of pyrethroid derivatives on autophagy mediated signaling and impact of different inhibitors on Pyrethroid derivatives-mediated autophagy. (**A**) and (**a**, **a-1**) Western blots were performed as described above. For this, THP1 cells were treated with Allethrin (100 μM) for the indicated time points. Cell lysates were then subjected to Western blot analysis for the indicated proteins. Blots were scanned and quantified using Image J. pAkt, pERK1/2, Beclin 1, LC3, p62 were normalized for loading using Akt, ERK2 or Tubulin. (**B**). Cells were pretreated with U01206 (10 μM for 1 h) and bafilomycin A1 (BafA1, 10 nM for 1 h), followed by stimulation with Allethrin (100 μM) or Prallethrin (100 μM) for 90 min and 24 h.phospho-ERK1/2 and Beclin 1 were determined by Western blot analysis. Representative blots are shown in the left column. *N* = 3. ^**^ *p* < 0.01; ^***^ *p* < 0.001; ^****^ *p* < 0.0001.

To explore the prominence of Akt/ERK in induction of autophagy, we examined using human THP-1 cells. Treatment with Allethrin substantially increased the proportion of phospho-ERK1/2, Beclin 1, LC3B, with a prominent decline in p62 and phospho-Akt levels shown in (Figure 5A, a and a-1). Indicating that autophagy response noticed depends on both Beclin 1 and activation of ERK. Conversely, pretreatment of cells with U01206, a selective ERK inhibitor significantly decreased phosphorylation of ERK1/2 and similarly pretreatment of the cells with bafilomycin A1, an autophagosome-lysosome fusion inhibitor significantly reduced Beclin 1 levels (Figure 5B).

### Pyrethroids induces caspases activity

**Figure 6:**
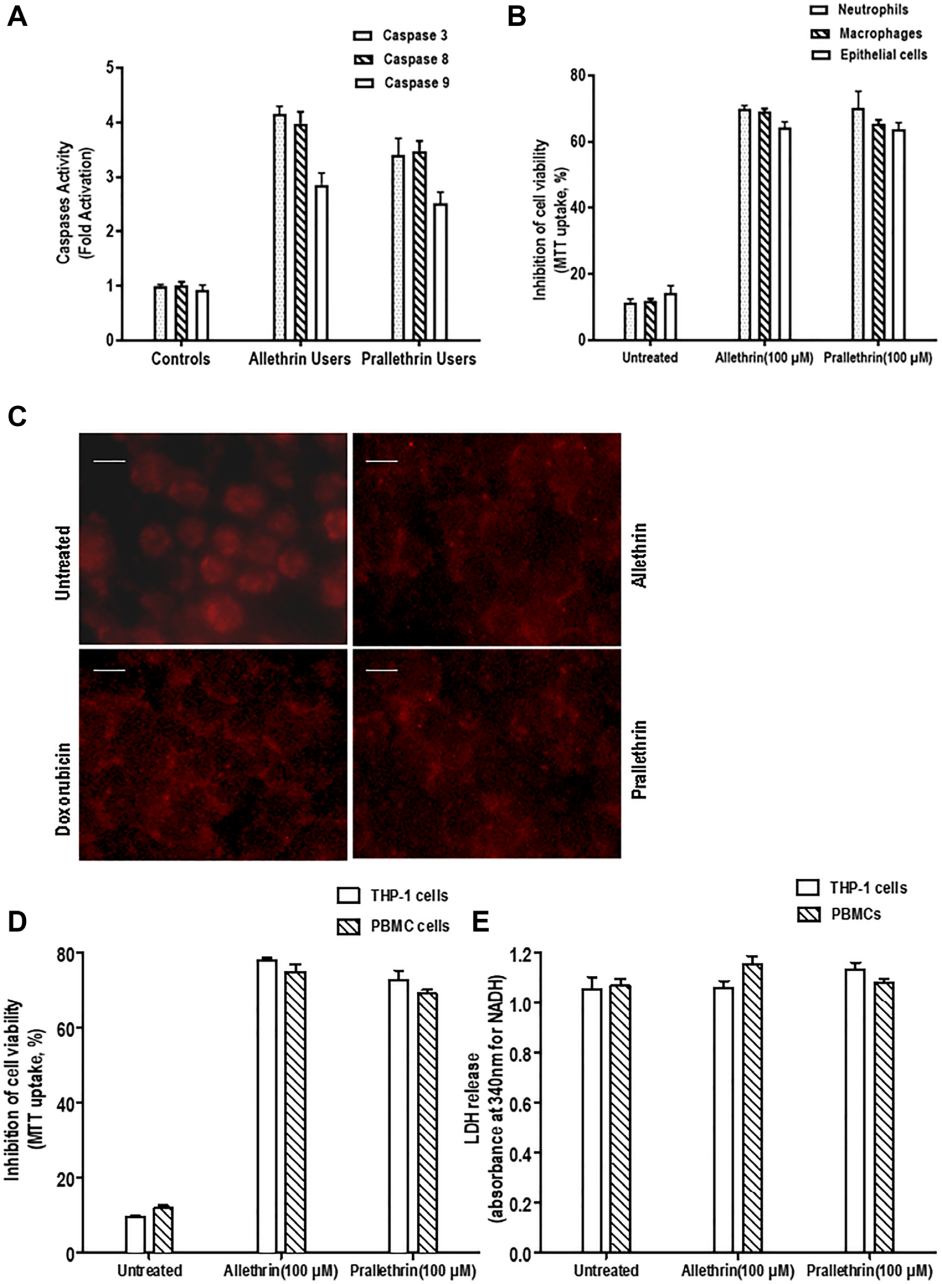
Effect of pyrethroid derivatives on caspase activity and cell death. (**A**). Caspase activity analysis in allethrin and prallethrin exposure users and healthy control PBMCs cells. Cells were washed with PBS and allowed to rest 24 h. Caspase 3, 8, and 9 were detected from whole cell extracts using colorimetric substrate. The absorbance was taken at 405 nm and results represented in fold activation of caspases. (**B**). Effect of Pyrethroid derivatives in different human primary cells: Isolated neutrophils, epithelial cells and differentiated macrophages were incubated without or with Allethrin (100 μM), or Prallethrin (100 μM) for 24 h. Cytotoxicity was assayed and mean absorbance of triplicate samples were calculated and indicated as inhibition of cell viability considering untreated cells as 100% cell viability. (**C**). Nuclear fragmentation was detected from Allethrin (100 μM), or Prallethrin (100 μM) and Doxorubicin for 24 hr treated THP-1 cells by propidium iodide staining. The fluorescence of stained cells was visualized under a fluorescence microscope (scale bar, 15 μM). (**D**). THP-1 cells and PBMCs were incubated without or with Allethrin (100 μM), or Prallethrin (100 μM) for 24 h and assayed for cell viability using MTT dye. The results represented as inhibition of cell viability.

Pyrethroid derivatives-related apoptosis was measured by the various caspases activation. Pyrethroid derivative users and controls PBMC cells whole cell extracts-(wce) were utilized along with the colorimetric paranitroaniline (pNA) substrates and were quantified for caspase 3, 8, and 9. Pyrethroid derivative users had increased all these caspases activities but not in controls (Figure 6A), suggesting pyrethroid derivatives mediates cell death in PBMC cells.

### Pyrethroids induces cytotoxicity and not necrosis in different human primary cells

To detect the pyrethroid derivatives and by external treatment in *in-vitro* human primary cells mediated cytotoxicty was analyzed. In pyrethroid derivative users over control PBMC cells the maximum inhibition of cell viability (60%) was noticed. Also, Pyrethroid derivative treatments in THP-1 and PBMC cells showed at most inhibition of cell viability (65%) was noticed. To validate the apoptosis effects for Pyrethroid derivatives at same concentration derived from the healthy human samples as Neutrophils, Macrophages and Primary Epithelial cells were tested. Similar results at this concentration was noticed where the maximum inhibition of cell viability (65%, 62%, 60%) was noticed. Cell viability was reduced in pyrethroid derivative users and treated cells indicating the cytotoxic effects of pyrethroid derivatives in the human primary cells (Figure 6B–6D).

## DISCUSSION

Pyrethroid derivatives and related products exposure have revealed to intensify various ailments and adverse health consequence in the different age groups, especially in hematological, respiratory disorders and immune related patients. In-depth gaining molecular mechanism for the detrimental impacts due to the accumulation of pyrethroid derivatives or its effects is necessary for future effective therapy. Autophagy (self-eating), is mostly termed as self-consumption process or mechanism in which cells strive to dispose unused contents internally and replenish this waste as source of the energy. Recent events divulge a pivotal role for dysfunction of autophagy in the hematological cancers, immune related disorders may get activated due to autophagy mechanism. We tracked the association of pyrethroid derivatives in autophagy or apoptosis, detection of the comprehensive mechanisms between both might facilitate to regulate pyrethroid derivatives associated deleterious effects. This study provides clues that the role of pyrethroid derivatives in autophagy and apoptosis mediated cell signaling is involved. Pyrethroid derivatives have been shown to aggravate several ailments and toxic effects particularly noteworthy in the neurotoxic, hepatotoxic, cardiotoxic, nephrotoxic, immunotoxic, and behavioral patterns of pyrethroids on human-subjects in different age group of people and hematological or immune related patients. Better spectrum studies of the molecular or cellular mechanism of these detrimental effects should be emphasized for the future effective therapy. We used pyrethroid derivatives, allethrin and prallethrin for current research studies; and to study their action or effects of pyrethroids, toxic doses utilized in the human primary cell lines and other cells were infinite range of physiological concentration [16, 28, 29, 30]. For the selective recruitment of monocytes, neutrophils, and lymphocytes, expression of chemokines play a significant role and also for enhancing chemotaxis through stimulation of G-protein-coupled receptors. MCP-1/CCL2, (Monocyte chemoattractant protein-1) is distinctive among the other chemokines that synchronize migration and infiltration of monocytes/macrophages. Dual CCL2 and its receptor CCR2 well denoted to be up regulated further indulge in different disorders [31]. Chemokine expression is associated with these diseases in the milieu of necrosis in tissue, acute inflammation, and inflammatory cytokine release. Perceive better elements moderating MCP-1 trigger, we studied the involvement of pyrethroid derivatives in the healthy and pyrethroid exposure user samples. In both allethrin and prallethrin exposure user samples substantially induced Ccl2 mRNA expression and also the protein levels over healthy controls. Earlier studies have suggested the role of Ccl2 induced levels and reactive oxygen intermediate generation during the oxidative stress conditions. Studies have demonstrated that ROS is involved in various cellular events like, motility of cell, cell differentiation or the cell-cycle progression and signaling of growth factors [32, 33]. Persistent or chronic exalted levels of ROS inside a cell have been ascribed to disorders, inclusive of inflammatory or immune related or lung disorders, CLL-chronic lymphocyte leukemia, AML-acute myeloid leukemia [34], etc., also in these disorders, superoxide dismutase and erythrocyte catalase like enzymes are diminished. Such deficiencies in enzymes lead to the accumulation of ROS, including superoxide (O2−), hydroxyl (OH•), along with nonradical species like hydrogen peroxide (H_2_O_2_) and hypochlorous acid (HOCl) [35]. Both allethrin and prallethrin exposure user samples had an increased magnitude of the ROI-reactive oxygen intermediate, associated cell death that is neither relied over reactive oxygen intermediate as noticed in the Hoechst staining. Further treatment with ROS scavenge (N-acetyl-cysteine) abolished up regulation levels. Previous studies have shown that ROS at elevated levels can recur abet polyunsaturated fatty acids of lipid membranes further enhance lipid peroxidation. Due to oxidative stress the reactive intermediates generated, can modify the membrane bilayers and tenent lipid peroxidation of the polyunsaturated fatty acids (PUFA) [36]. Lipid peroxidation along with wreckage of lipids, also the generation of reactive compounds elicit to alterations in the permeability and fluidity of the membrane lipid bilayer also can drastically vary the integrity of cells [37]. Pyrethroid derivative exposure user samples evaluation showed inhibited membrane bound enzymes and altered membrane fluidity in human platelet membrane and a substantial increment in membrane cholesterol (C) and lessen in phospholipid (P) contents with nil notable turnaround in the protein constituents in the platelet membranes were observed over the healthy controls. Pyrethroid derivative exposure user samples had induced levels of lipid peroxidation and induced binding activities of transcription factors like CEBP-β and NF-AT. The transcriptional factors CEBP-β and NF-AT are well-known to regulate various cellular events. Beclin1 as well as LCBs play a role in autophagy [38–40]. Pyrethroid derivatives, allethrin and prallethrin enhanced autophagy prospectively over the familiar inducers such as TNF or doxorubicin, manifested with increment in autophagy markers such as beclin1 and LC3B in fluorescence-labeled autophagosomes [18, 41–44]. The results also suggest BECN1 up regulation with preferential LC3B-II accumulation and significant p62 degradation. Bafilomycin A1, known for noted inhibitor of autophagosome maturation, [45, 46] effectively shielded the pyrethroid derivatives-mediated autophagosome from degradation, else indicating pyrethroid derivatives-mediated autophagy. Studies have shown that alterations in the membrane fluidity and related enzymes can regulate cellular Ca^2+^ homeostasis [47]. Due to these alterations in this membrane activity, it expedites to elevations in intracellular free Ca^2+^ level. Enhanced Ca^2+^ increases calcineurin activity. Calcineurin, known serine/threonine phosphatase dephosphorylates its substrate protein NF-AT in the cytoplasm. Pyrethroid derivatives exposure user samples induced calcineurin through enhanced Ca^2+^ level. Multidomain signaling scaffold protein p62, cohere to LC3B and is gathered, next step by decay in autophagosomes [39]. Pyrethroid derivatives inhibited the p62 protein expression levels. Autophagy related-molecular mechanism studies have shown to display the regulative molecules that comply autophagy are diversified, like ERK1/2, AMP kinase, class I and class III PI3K, Akt, mTOR and others [48]. ERK1/2-Extracellular signal-regulated kinase signaling too modulates autophagy as well as the lysosome genes expression, and proven to trigger autophagy by interacting with LC3. Pyrethroid derivatives exposure user samples treatment didn’t alter the phospho-Akt levels but had greatly amplified the levels of phospho-ERK1/2. Both in the *in-vitro* and *in-vivo,* ERK1/2 binds a docking site within DAPK1’s death domain and phosphorylates DAPK1 Ser735 within cytoskeletal binding region stimulating DAPK1 catalytic activity. Calcium/calmodulin-regulated (CaM-regulated) protein kinase, DAPK1 stimulates death signaling in regards to IFN-γ, TNF-α, or other cytokines. DAPK1 is known to be well studied in the autophagy, immune response to inflammatory signals and also in proliferative signaling [49]. Studies have been mentioned that Bim operates as a molecular link midst autophagy and apoptosis. Upon death stimuli response, Bim detaches from the dynein light chain 1 and later instigates BAX/BAK-mediated mitochondria-dependent apoptosis. Normally autophagy stops the inference of apoptosis, and apoptosis-linked caspase activation closes off the autophagic process. But, in selective instances, autophagy or autophagy-pertinent proteins might facilitate to trigger apoptosis or necrosis and autophagy has been presented to diminish the cytoplasm exceedingly, for autophagic cell death [50]. Here in we observed pyrethroid derivatives elicited proapoptotic genes, DAPK1/Bim expression levels and in turn activated the caspases and leading to cell death. Pyrethroid derivatives exposure user samples induced cell death in different human primary cells - PBMCs, PRP cells, Neutrophils, Epithelial cells and differentiated Macrophages. The current research study shows a critical and a central role of autophagy in the signaling cascades, suggesting its major role in pyrethroid derivatives- induced apoptosis or cell death.

Autophagy and Apoptosis regulate the yield of proteins inside cells and various stress assisted pathways systematically evoke autophagy, and apoptosis inside same cell. The converse between autophagy and cell death pathways impacts the usual clearing of dying cells and also in dead cell antigens, immune recall or remembrance. So, the alliance linking of autophagy and apoptosis in hematological malignancies or the related immune mediated disorders has a very prominent pathophysiological consequences. Hence, our study findings has tremendous importance and helps in understanding the pyrethroid derivatives modus operandi of trigger and their detrimental impacts. Future studies need to be goaled by scheming design of relevant drugs-therapeutics to modulate above discussed signaling pathways and to negate the deleterious impacts.

## MATERIALS AND METHODS

### Materials

Except mentioned or else, entire chemicals and anti-tubulin antibody were procured from Sigma-Aldrich (St. Louis, MO, USA). TRIzol, from Invitrogen. FBS, DMEM, cell culture reagents were bought from Life Technologies. Caspase substrates (Ac-DVED-pNA, Ac-ITED-pNA, and Ac-LEHD-pNA), DAPI, Fura-2AM were procured from Molecular Probes (Eugene, OR, USA). Antibodies procured from Cell Signaling Technology (Beverly, MA, USA).

### Methods

#### Subjects for study

In this study volunteers were considered utilizing either-or Jet^®^ mosquito repellent coils or mats, duo from Godrej *Sara Lee Ltd.,* Mumbai, INDIA. Composition of coils with (w/w) 0.1% *d-trans* allethrin, 52.9% wood flour, 35% coconut shell powder, 12% starch, and the mats comprised (w/w) 1.6% *d-trans* prallethrin and 98.4% applicable ingredients as provided by the entrepreneurs. The outcome of pyrethroid insecticide is either-or by burning coil or subjecting mat in the readily accessible electronic device. Study subjects were aware to get exposed to allethrin or prallethrin for relatively 8 h/day instead of 10 h/day, subjects with nil history of exposure to alike pyrethroids. Three-(3) groups, every group comprising of 10 male volunteers aged midst 35–45 years, considered for current study were: Group I, controls whom never used mosquito repellents; Group II, subjects allethrin exposure; Group III subjects prallethrin exposure shown in (Table 1). The study volunteers were thoroughly educated regarding experimentation and the individual written consent were documented or collected. Current research study was approved by institutional ethical committee at Sri Krishnadevaraya University, AP India. In the presenting study involved with human subjects complies with the Declaration of Helsinki. Overnight fasted subjects, blood samples from were collected for the current study. The research study volunteers chosen in the current study were divest of illness or any chronic disorders also were mostly teetotalers with nil smoking habit, also were not using tranquillizers, drugs and anaesthetics.

### Blood collection and platelets isolation

Collection of blood from human volunteers was done by venipuncture midst of 7 AM to 10 AM. Later, blood of 10 ml volume was taken along (ACD) acid citrate dextrose anticoagulant solution in ratio of 9:1. To obtain PRP, centrifugation at 160 g for 10 min was done using anticoagulated blood. The obtained PRP was centrifuged at 160 g to expel red blood cells. Followed by then to pellet out platelets, PRP was centrifuged at 300 g for 5 min. The washing steps were continued till the suspension was rid of erythrocyte and purity was verified under microscopic examination. Until the further analysis, platelet storage buffer containing 0.109 M NaCl, 4.3 mM K_2_HPO_4_, 16 mM Na_2_HPO_4_, 8.3 mM NaH_2_PO_4_ and 5.5 mM glucose, pH 7.5 was used wherein the platelet pellet was suspended in and stored at 4^°C^.

### Cell lines

The human primary cells-Peripheral blood mononuclear cells (PBMC), Neutrophils, Macrophages were derived from fresh human blood using Ficoll-hypaque centrifugation protocol from healthy and patient samples. Interior side of lower lip was scrapped for the epithelial cells and suspended in medium. Derived cells were considered as primary epithelial cells. THP-1 cells were procured from ATCC. DMEM medium containing 10% FBS, penicillin (100 units/ml), and streptomycin (100 μg/ml) was used to culture the cells. Cells used for the studies were free from mycoplasma contamination and were tested using the gen-probe mycoplasma detection kit (Fisher Scientific).

### Isolation of platelet membrane

The isolation of platelet membrane was adopted from [51], platelet suspension with the equal volume and Triton X-100 lysis buffer held in microfuge tubes, mixed by inversion. Immediately, the clear suspension of platelets was centrifuged at 4°C for 2.5 h at 100000 g. Decant the supernatant from tube and the translucent platelet membrane pellet was cautiously retained and utilized for the measurement of lipids and further parameters.

### Estimation of lipids in platelet membrane

Lipids derived of platelet membrane were obtained by method refers as [52], 2 mg of protein/ml content from membrane preparation was used to mix the chloroform/methanol mixture (2:1, v/v) with a ratio of 1:9 (v/v). Homogenization of the solution was done at moderate speed, wherein organic lipid layer was cautiously removed and evaporated to dryness in a conical flask. Later with a familiar volume of chloroform/methanol mixture, the lipid was dissolved. As per the mentioned previous method [53] after [54] perchloric acid digestion, phospholipids in total were measured in terms of inorganic phosphorus. By utilizing ferric acetate/uranyl acetate reagent [55], the platelet membrane cholesterol was measured.

### Lipid peroxidation in platelets

The level of lipid peroxidation was quantified for generation of malondialdehyde (MDA) as per mentioned protocol [56]. Platelet membrane with one ml was collected in test tube, with the addition of 2 ml of reagent (15% w/v TCA, 0.375% w/v TBA and 0.25N HCl), then placed in boiling water bath for 15 minutes and all components were permitted to cool, followed by centrifugation at 1000 g for 10 minutes. In a different test tube supernatant was transferred and the sample absorbance using a UV/Visible spectrophotometer measured at 535 nm against the reagent blank assuming the molar extinction coefficient to be 1.56 × 10^5^.

### Assay of platelet membrane-bound enzymes

Total ATPase activity was studied as per the stated method [57]. ATP as substrate was taken along with Na^+^, K^+^ , Mg^2+^, and Ca^2+^ions. As per method [58] Na^+^, K^+^-ATPase activity was quantified in the presence of Na^+^ and K^+^ ions. Mg^2+^-dependent ATPase level was measured by the protocol [59] and Ca^2+^-dependent ATPase activities were measured using method [60] taking substrate as ATP in the proximity of Mg^2+^ and Ca^2+^ ions. Nanomoles of phosphorus released/minute per 1 × 10^5^ platelets were denoted for entire activity of ATPases assays.

### Fluorescence measurement

Fluorescence measurements of platelet membranes and lipid extracts were used and executed on the spectrofluorometer. Fluorescence anisotropy (*r*) quantifications in a steady-state utilizing the excitation and emission wavelengths at 360 nm and 430 nm, for DPH and pyrene were documented. Fluorescence anisotropy (*r*) degree was estimated as per equation [61]. For the assay final protein concentration was 0.4 mg/ml, the probe concentration was 10^−6^M. On the lipid extracts fluorescence measurements were executed as per method [62] and was normalized to same content of proteins (0.4 mg/ml). Overall the samples were suspended in 10 mM Tris pH 7.4 and then the quantifications executed at 25°C.

### MDC Staining

Cells after treatments were rinsed thrice with wash buffer-PBS (pH 7.4) and then using MDC were stained for 10 min at 37°C. Later intracellular MDC have been quantified using the fluorescence photometry/fluorimetry (Excitation-380 nm and Emission-525 nm) detailed previously [63]. Alike, treated cells were utilized by fluorescence microscopy for qualitative analysis of autophagy. With fluorescence intensity enhancement in the MDC-stained autophagosomes, autophagy was determined and measured. Autophagy level is denoted as-fold increment per relative fluorescence units in these experiments.

### Immunofluorescence staining

Cells treated were rinsed gently thrice followed by fixation using 4% paraformaldehyde for 15 min at 37°C, subsequently permeabilization with 0.1% Triton-mediated at 30°C for 15 min. Then gently rinsed with PBS thrice, next 1% BSA was used at RT for 30 min for blocking and relative primary antibody for incubation at 4°C for 90 min. Followed by this step, PBS was used for washing thrice and incubation with secondary antibody in the dark for 90 min. The slide post rising and air-drying, DAPI was added followed by cover slip was mounted gently to avoid bubbles.

### Cytokine/chemokine measurements

ELISA kits from eBiosciences, Inc. as mentioned earlier [64] were utilized to quantify cytokines and chemokines levels using plasma samples.

### Determination of caspases activity

Different caspases activities were measured as elaborated in the study priorly [65], utilizing colorimetric (paranitroaniline conjugated) caspase substrates in accordance to the manufacturer’s protocol Calbiochem (San Diego, CA, USA).

### RNA extraction and real-time Q-PCR

Human PBMCs cells were prepared 24 hours post-treatments, and then total RNA was collected utilizing Qiagen’s RNeasy Mini kit. 1 μg of RNA was utilized for reverse transcription with Promega cDNA synthesis kit. The expression of Ccl2, DAPK1, Bim, Beclin-1, LC3 and HPRT was executed as mentioned [65]. using Real-time Q-PCR. IDT DNA Technologies supplied the primers. The mentioned primers were used: Ccl2-Forward: ACT CTC GCC TCC AGC ATG AA; Reverse: TTG ATT GCA TCT GGC TGA GC; DAPK1-Forward: CAG TGT TGT TGC TCT AGG AAG; Reverse: GGG ACT GCC ACA AAT GAT GAG; Bim-Forward: TGG CAA AGC AAC CTT CTG ATG; Reverse: GCA GGC TGC AAT TGT CTA CCT; Beclin-1Forward:GAG GGA TGG AAG GGT CTA AG; Reverse: GCC TGG GCT GTG GTA AGT; LC3-Forward:GAG CAG CAT CCA ACC AAA; Reverse: CGT CTC CTG GAG GCA TA; HPRT-Forward: AAG CCT AAG ATG AGC GCA AG; Reverse: TTA CTA GGC AGA TGG CCA CA. ABI fast 7500 (Applied Biosystems) was used for entire Real-time Q-PCR and total genes were normalized to HPRT.

### Intracellular Ca^2+^measurements

Intracellular Ca^2+^ concentration [Ca^2+^]i was accessed with the fluorescent Ca^2+^ indicator fura-2-acetoxymethyl ester (Fura-2/AM) as mentioned [66]. THP-1 derived macrophage cells were cultured in T75 flasks till they were confluent, next step with starvation of serum for 2 h then loaded with 2 μM Fura-2/AM for 45 min. Cells were later rinsed, followed by trypsinization. Then the trypsinized cells were utilized for the assay. The cells were resuspended in the presence of Ca^2+^ either in the Ca^2+^-consisting HEPES-buffer (138 mM NaCl, 5 mM KCl, 1 mM MgCl_2_, 2 mM CaCl_2_, 10 mM glucose and the 10 mm N-2-hydroxyethylpiperazine-N′-2-ethanesulfonic acid (HEPES)/NaOH, pH = 7.4), or in the Ca^2+^ free buffer, which comprised of 0.1 mM EGTA instead of CaCl_2_. Before and after corresponding treatments, Fura-2 fluorescence intensity quantified spectrophotometrically in the stirring cuvette at excitation wavelengths of 340 and 380 nm and an emission wavelength of 510 nm. Fura-2 fluorescence (340/380 nm) ratio values were used to represent the data.

### Calcineurin activity assay

Following various treatments, cells (2 × 10^6^) were collected and the extracts were subjected through sephadex G-25 column to remove the free phosphate and proteins fractions have been collected collectively [67] to investigate the calcineurin activity assay. Later 10 μg protein extract with 25 μl of 2× assay buffer (200 mM NaCl, 100 mM Tris [pH 7.5] and 12 mM MgCl_2_, 1 mM CaCl_2_) was incubated. Mixture was treated without or with RII phosphopeptide (5 μM) for 10 min at 30°C. The reaction was terminated by adding 100 μl of Malachite green mix (3 volume of 0.045% Malachite green and 1 volume of 4.2% ammonium molybdate in 4N HCl) and then allowed for 30 min incubation at 30°C. The absorbance read out was done at 660 nm. Inorganic phosphate release from total protein and unstimulated cells value were considered as one fold and quantified for the calcineurin activity (fold).

### Measurement of reactive oxygen species (ROS)

A black 96 well plate with clear bottom was used to plate or seed the cells and then treated with Allethrin (100 μM), Prallethrin (100 μM), DMSO (100 μM) and NAC (10 μM) for 0 hr, 1 hr, 3 hr, 6 hr, 9 hr, 12 hr and 24 hr. Following all the treatments 100 μl of 10 μM 2’, 7’-dichlorodihydrofluorescein diacetate (H_2_DCFDA) was diluted in PBS was added to each well. ROS related fluorescence levels were quantified kinetically taking a fluorescence plate reader at excitation 494 nm and emission 525 nm, at room temperature. After ROS measurement, data was normalized according to cell number using a Hoechst 33342 stain at excitation 350 nm and emission 461 nm on each well.

### Molecular docking studies

Allethrin and Prallethrin were taken for virtual screening towards the selected target receptors to study their molecular interaction and inhibition mode by docking simulation. Ligand molecules, Allethrin and Prallethrin sourced through PUBCHEM and then partial charges were adjoined in PRODRG server. The crystal structures of CEBP-β (PDB id: 1GU4) and NFAT (PDB id: 2o93) have been obtained from Protein Data Bank [68]. All non-protein molecules were eliminated, before commencing the docking simulations and for the other atoms locations only needed location was retained. Auto Dock4.2 docking program was applied for each ligand and was docked into the energy minimized preceptors. In the present study Autodock has been applied exclusively used along with Lamarckian genetic algorithm (LGA) [69]. Atomic affinity potentials of individual atom type in ligand are determined utilizing the grid based maps. Ligand conformation in docking simulations was evaluated by grid based energy. The ligands situated in auto grid concealed grid map dimensions 60Å × 60Å × 60Å with grid spacing of 0.375Å. For all the docking simulations population size and docking runs have been marked to 300 and 100 appropriately. Further docking parameters have also positioned as default values for all the diverse algorithms. The result findings for docking were graded by the binding energy (kcal/mol) and inhibition constants (Ki).

### Western blot analysis

Whole-cell extracts (WCEs) were extracted after various treatments and then for the current experiments 50 μg of the protein was taken. The same gel using stripping buffer was stripped and utilized to detect or check for tubulin.

### Cytotoxicity assay

MTT dye was used to for the cytotoxicity assay [70]. Cells approximately (10^4^ cells per well of 96-well plate) have been treated using test sample for 24 h at 37°C. Followed by, to each well 100 μg MTT dye was added. Post incubation for 2 hr, then the cells were solubilized. A multi scanner auto reader (Biorad), taking extraction buffer as a blank was used for absorbance read out at 570 nm.

### Necrosis assay (LDH release measurement)

LDH, a cytosolic marker was assayed to estimate cell necrosis for allethrin or prallethrin treated cells from the culture supernatant. Culture supernatant was incubated using substrate solution comprising 230 mM sodium pyruvate and 5 mM NADH in 0.1 M phosphate buffer (pH 7.5). After incubation absorbance was quantified at 340 nm.

### Statistical analysis

Entire data represented are as the mean ± SEM from one experiment of triplicate samples. By using Student’s *t* test, two group comparisons were done and ANOVA with the post-Bonferroni test were performed for comparisons of more than two groups. Then, unpaired student’s *t* test or one-way analysis of variance next by applicable post hoc test (Tukey’s multiple comparison test) using GraphPad Prism5, were taken for the statistically significant differences. *p* < 0.05 were accounted to be noteworthy.

### Consent for publication

Followed as per the journal guidelines towards the publication.

## SUPPLEMENTARY MATERIALS


