# Supplementary material for: The role of pyrethroid derivatives in autophagy and apoptosis crosstalk signaling and potential risk for malignancies
Source: Oncotarget. 2022 Dec 17;13:1323–40. doi: 10.18632/oncotarget.28328 (PMC9760267; doi:10.18632/oncotarget.28328)
Supplement: Supplementary file 1 [file oncotarget-13-28328-s001.pdf]

The role of pyrethroid derivatives in autophagy and apoptosis crosstalk signaling and potential risk for malignancies

SUPPLEMENTARY MATERIALS

Supplementary Table 1: Physicochemical properties calculated for compounds using molinspiration server

Compound	log P[a]	TPSA[b]	*n* atoms	MW[c]	*n* ON[d]	*n* OHNH[e]	*n* rotate[f]	Volume
Allethrin	4.73	43.38	22	302.41	3	0	6	303.86
Prallethrin	4.24	43.38	22	300.40	3	0	5	298.39

[a]Log P (Lipophilicity): No greater than 5; [b]TPSA (Polar surface area): No greater than 140 $Å^2$; [c]MW: 180-500 Daltons; [d]n ON (H-bond acceptors): should be less than 10; [e]nOHNH (H-bond donors): should be less than 5; [f]No. of rotatable bonds may 10 or fewer.

Supplementary Table 2: ADMET predicted profile for compounds obtained from preADMET web server

Compound	Human Intestinal Absorption (%)[a]	*In vitro* Caco-2 cell permeability (nm/second)[b]	*In vitro* MDCK cell Permeability (nm/second)[c]	*In vitro* plasma protein binding (%)[d]	*In vivo* blood brain barrier penetration (C.brain/C.blood)[e]	Pgpinhibition
Allethrin	97.98	44.52	5.22	90.70	0.39	Inhibitor
Prallethrin	97.99	44.52	5.22	90.70	0.39	Inhibitor

[a]Human intestinal absorption: 0–20% (poor absorption), 20–70% (moderate absorption), 70–100% (well absorption). [b]*In vitro* Caco-2 cell permeability (nm/second): The values are <4: low permeability,4–70: middle permeability, >70: high permeability. [c]*In vitro* MDCK cell permeability (nm/second): The values are <25: low permeability, 25–500: middle permeability, >500: high permeability. [d]*In vitro* plasma protein binding (%):The values <90%: weak binding and >90%: strong binding. [e]*In vivo* blood-brain barrier penetra tion (C.brain/C.blood): The values <0.1: low absorption, 0.1–2.0: middle absorption, >2.0: higher absorption.

Toxicity results of compounds:

Parameter	Allethrin	Prallethrin
Ames_test	mutagen	mutagen
Carcino_Mouse	positive	positive
Carcino_Rat	positive	positive
hERG_inhibition	low- risk	low-risk
TA1535_NA	negative	positive

Supplementary Table 3: Molecular docking interaction results of CEBP-β and NF-AT with allethrin and prallethrin

Protein	Ligands	Lowest binding energy (Kcal/mol)	Inter molecular energy (Kcal/mol)	Inhibition constant (μM)	Amino acids involved in hydrogen bond formation
CEBP-β	Allethrin	−4.68	−5.33	373.92	Thr-299, Lys-302, Val-303, Lys-302, His-301
	Prallethrin	−4.82	−5.29	293.44	Gln-300, Lys-302, Asn-296, Glu-298, His-301,Val-303
NF-AT	Allethrin	−4.5	−5.32	502.82	Arg-572, His-419, Pro-436, Lys-417, Arg-421, His-420
	Prallethrin	−4.48	−5.2	517.24	Arg-572, His-419, Glu-568, Val-542, Leu-499, Gln-571

Docking energies are as determined by AutoDock.
